# Values in Time Discounting

**DOI:** 10.1007/s11948-017-9950-y

**Published:** 2017-08-15

**Authors:** Conrad Heilmann

**Affiliations:** Erasmus Institute for Philosophy and Economics (EIPE), Faculty of Philosophy, Erasmus University Rotterdam, P.O. Box 1783, 3000 DR Rotterdam, The Netherlands

**Keywords:** Time discounting, Discount rate, Value-free ideal, Stern review, climate economics, Philosophy of economics

## Abstract

Controversies about time discounting loom large in decisions about climate change. Prominently, a particularly controversial debate about time discounting in climate change decision-making has been conducted within climate economics, between the authors of Stern et al. (Stern review on the economics of climate change, 2006) and their critics (most prominently Dasgupta in Comments on the Stern review’s economics of climate change, 2006; Tol in Energy Environ 17(6):977–981, 2006; Weitzman in J Econ Lit XLV:703–724, 2007; Nordhaus in J Econ Lit XLV:686–702, 2007). The article examines the role of values in this debate. Firstly, it is shown that time discounting is a case in which values are key because it is at heart an ethical problem. Secondly, it is argued that time discounting in climate economics is a case of economists making frequent and routine references to ethical values and indeed conduct ethical debates with each other. Thirdly, it is argued that there is evidence for deep and pervasive entanglement between facts and values in the prevalent methodologies for time discounting. Finally, it is argued that this means that economists have given up the ‘value-free ideal’ concerning time discounting, and discussed how the current methodology of time discounting in economics can be improved.

## Introduction

Few scientific topics are more controversial than climate change. Few topics in economic theory are more contentious than time discounting. In the emerging field of climate ethics and economics, they come together. This article discusses the role of values in time discounting in this field, taking a philosophy of science perspective.

Time discounting lowers the value of costs and benefits in the future, relative to how far in the future they are: usually, any present costs and benefits are assigned the full weight, and any future costs and benefits are assigned weights in decreasing fashion, with such weights being typically between 1 and zero. Discounting weights of this kind are routinely included in economic analyses. Depending on how drastically these weights decrease for future times, the impact of time discounting on decision-making can range from being negligible, to swinging a decision completely. Consider the following simplified case, where a decision has to be taken whether to protect the environment today at a cost of 40 Million Euros or face the costs of 45 Million Euros for clean up in 10 years. If there are no further considerations, then one should decide to protect the environment today. If, on the other hand, discounting is employed, then the discounted value of the future clean-up costs could be much smaller than today’s protection costs. If one assumes a discount rate of 2% per year, the discounted clean-up costs of 45 Million Euros in 10 years time amount to 37 Million Euros today.^1^ This may change the decision.

The example is oversimplified in many ways, and deliberately so. It does not: speak of opportunity costs for the money used today, incorporate any details of the financial market, consider any alternative courses of action, investigate whether costs in 10 years can be adequately valued, consider whether environmental protection and costs of clean-up can be both captured adequately and compared in monetary terms, ask what sort of uncertainty goes into assuming it is actually possible to clean up the environment, ask what sort of uncertainty is associated with the effectiveness of measures to protect the environment today, look into the precise method of calculating the discounted value, explain the derivation of the discount rate that determines the discount factor for any given time, consider political questions about making decisions for people so far in the future, consider the irreversibility of the effects involved in failing to protect the environment at a certain point in time, take into account ethical questions that arise when unborn people may suffer the consequences of decisions taken by those alive today, and so forth. The list could easily go on for another page before even invoking any of the additional complexities that come with the topic of climate change more specifically. Intertemporal decisions, and especially intergenerational ones, are complex and multi-faceted.

The complexity of intergenerational decision-making is one reason for why time discounting is an important topic. As seen in the example, time discounting is, at least on the face of it, an astonishingly simple tool. All it does is to produce one weight per time point, and that weight expresses the relative significance of what happens at that time from the viewpoint of the present. When chosen carefully, time discounting can thus ‘overturn’ many other factors in a decision-making process. This deceptively simple approach to intertemporal decision-making has, not unsurprisingly, lead to prolonged debate amongst philosophers, social scientists, and economists alike. The case of climate economics and climate ethics is thus an interesting case from a *methodological perspective*. From the perspective of climate economics and ethics, though, time discounting is a contested tool that different proponents in economic and ethical debates have attempted to deputize for their respective positions. As the earlier example suggests, the magnitude in which time discounting can influence decision-making dwarfs any uncertainty that climate change scientists attach to their projections. The stakes could hardly be higher.

This article adopts the viewpoint of a philosopher of science to examine the methodology of discounting, and in particular the controversies about discounting in climate economics. Four observations are made. In the first section, entitled "Time discounting in economics", it is shown that time discounting is a case in which values are key because it is at heart an ethical problem. Secondly, in the section entitled "Values and time discounting in climate economics", it is argued that time discounting in climate economics is a case of economists making frequent and routine references to ethical values and indeed conduct ethical debates with each other. One particular episode in climate ethics and economics is focused on, in which time discounting has featured quite prominently: the publication of the ‘Stern Review’ in 2006 and the debate about discounting it generated amongst climate ethicists and economists. A number of prominent economists and philosophers have reacted to the ‘Stern Review’, and in particular, the economists invested in the debate have focused on the use of time discounting in the recommendations of the review (most prominently Dasgupta 2006; Tol 2006; Weitzman 2007; Nordhaus 2007). The section entitled "Methodological values for time discounting" makes the remaining two observations. First, it is argued that there is evidence for prevalent methodologies of time discounting failing to separate facts and values. The separation of facts and values is however a strong, as well as contested, requirement. It is then argued that the prevalent methodologies of time discounting also fail to separate ethical and scientific judgements, which is a much weaker and less contestable requirement than the separation of facts and values. One of the implications of these observations is that it might be these two types of entanglements, which are inherent in the debates about time discounting in climate economics, which have contributed to it being such a vexed problem in the first place. Briefly, strategies of how to disentangle ethical and scientific judgements and how to improve the methodology of time discounting more generally are discussed.

## Time Discounting in Economics

### Time Preferences and the Social Discount Rate

Most accounts of time discounting imply the proposition that goodness evaluations of future prospects can be devalued relative to present and earlier ones. In economics, this is achieved by a time discounting function. In its most general form, such a time discounting function assigns numerical values to time points. Most common: such an assignment gives the present the unit value, and future times are given non-zero weights that are smaller than 1. More formally, for a non-empty set of time points T = {0, 1, …} that includes 0 as the present time point, a time discounting function D can be written as D:T → (0,1], where the mapping is decreasing and D(0) = 1. But how can such a time discounting function be derived, and how can assumptions about the decreasing values over time and the assignment of the full weight to the present be given a precise foundation?

In economics, there are two dominant answers given to these questions. One, especially relevant for individual decision-making, involves the concept of time preference. Another, more relevant for social and intergenerational decision-making, involves the concept of the social discount rate.

Let us start with the concept of time preference. In order to derive a discounting function, many economists invoke the concept of time preference. Time preference entails the assumption that individuals have a taste for experiencing positive events earlier rather than later, and negative events later rather than earlier. Frederick et al. (2002) review different conceptions of time preference, offered by Austrian economists in the 19th century, who initially proposed to think about intertemporal decision-making in this way. These economists thought, for instance, that individuals conceptualise the present as more ‘lively’ than the future (Rae), and that they ‘possess inadequate power’ to imagine the future (Böhm-Bawerk). Later, Pigou thought of time impatience as ‘a type of cognitive illusion’ and Ramsey thought it stemmed from a ‘weakness of imagination’ (for a richer historical account, see Frederick et al. 2002, p. 352–255).

Capturing time impatient attitudes to the future as a kind of taste allows economists to amend the usual framework of representing preferences by a utility function with *time preferences* to derive discounted utility. In standard rational choice theory, preferences over alternatives can be represented by a utility function, provided such preferences satisfy certain conditions (such as completeness, transitivity and continuity axioms). To capture intertemporality in rational choice theory, time preferences over alternatives that happen at a particular time can be represented by discounted utility, provided such time preferences satisfy certain conditions (such as completeness, transitivity, time impatience, stationarity, and continuity axioms). Initially proposed by Samuelson (1937), time preference has now become the cornerstone of models of intertemporal decision-making in economic theory (see Koopmans 1960; Lancaster 1963; Fishburn and Rubinstein 1982; Bleichrodt et al. 2008 for more recent accounts).

In a social and intergenerational context, time preference does not seem to be adequate, as it only refers to individual attitudes about the future. Social and intergenerational discounting is mainly conducted within the methodology of the so-called social discount rate. The social discount rate, often denoted r, is used to generate a discounting factor D in the following way: D = [1/(1 + r)]^t. Using this formula, a discounting factor for any point in the future is obtained which can then be applied to benefits that occur at that point in the future. The social discount rate is commonly taken to be a compound concept, reflecting a number of different motivations. Following Ramsey (1928), the social discount rate consists of several components: r = delta + eta * g, where delta is the pure time preference, eta is the elasticity of the utility of marginal consumption (the percentage change in welfare derived from a percentage change in consumption or income), and g the growth rate of per-capita consumption or income over time.

Economists have settled on methodologies for discounting the future that involve deriving a discounting function from either time preference alone (for individual decision-making) or from a social discount rate (for social and intergenerational decision-making). While there are also other methods to discount in economics for specific contexts, this article focuses on these two methodologies. Time preference, discounted utility, and the social discount rate are most widely adopted in economics. Indeed, the social discount rate is also the prevalent method for discounting in climate economics.^2^

### Time Discounting: An Ethical Problem

Time discounting functions—in both derivations—capture attitudes and judgements about the future. Let us now return to the initial thought about time discounting entailing that future goodness is weighted less than present goodness. From this perspective, it should be clear that time discounting entails an ethical value judgement. Indeed, in the philosophical literature, time discounting has been discussed as an ethical problem.

Rawls (1971, p. 259) channels Sidgwick when he says that ‘The mere difference of location in time, of something’s being earlier or later, is not a rational ground for having more or less regard for it.’ As early as in the Protagoras, the following statement can be found: ‘… if any one says: ‘Yes, Socrates, but immediate pleasure differs widely from future pleasure and pain’—to that I should reply: And do they differ in anything but pleasure and pain? There can be no other measure of them.’ There is thus a long and strong tradition in philosophy to view time discounting as ethically problematic.

Contrary to this view, there is also Derek Parfit, who in Reasons and Persons (Parfit 1984) maintains: ‘My concern for my future may correspond to the degree of connectedness between me now and myself in the future … since connectedness is nearly always weaker over long periods, I can rationally care less about my further future.’ While Parfit also advances arguments against social discounting in the same book, he does seem to suggest that this could be different for the case of individual time discounting.

The aim of this article is not to settle these ethical debates here. Rather, these positions illustrate that time discounting (with, or without, a connection to climate economics) is in essence an ethical problem. While stating that is bordering on the trivial, it is precisely the relation between the ethical nature of time discounting and the way in which this is reflected in the methodology of time discounting in economics that is of interest here. For instance, Ramsey (1928), concerning the social discount rate, seems to agree with viewing time discounting as an ethical problem, but also reaches an awkward conclusion concerning the methodology of time discounting: ‘It is assumed that we do not discount later enjoyments in comparison with earlier ones, a practice which is ethically indefensible […] we shall, however, … include such a rate of discount in some of our investigations.’ There is thus a considerable unease for Ramsey here, with both making an ethical judgement, but also in adopting a methodology in which ethical judgements seem to be inescapable.

Time discounting, being an ethical problem and thus involving value judgements, seems to be an important case to study for how economists deal with value judgements. A separation of facts and values has since long been upheld as a methodological ideal in economics (Dasgupta 2005). This provokes the following questions: how did more recent debates concerning time discounting, an ethical problem in and of itself, fare with regards to the separation of facts and values? How do the prevalent methods of time discounting reflect ethical values? Do economists engage in ethical debates concerning time discounting? Can analysing the role of values in time discounting help to better understand the debates in climate ethics and economics concerning discounting?

## Values and Time Discounting in Climate Economics

In order to answer the questions raised in the previous section, this section engages with climate economics more closely. In particular, it focuses on one episode in climate economics, that of the debates following the Stern Review (Stern et al. 2006). This is a prominent case that shows the important role of values in discussions about discounting in climate economics. Two observations will be made: firstly, it is shown that in debates about time discounting in climate change economics values are key, and secondly, it is shown that the *methodology* of time discounting entangles facts and values.

### The Stern Review and its Critics

In an intergenerational context, time discounting is the method of weighting costs and benefits to future generations less than those to the present one. It is widely employed in public policy decisions with a large time-scale. Famously, there is widespread disagreement about intergenerational discounting, in particular about its social scientific foundations and its conceptual and ethical justification. Recently, the controversy about intergenerational discounting has received renewed attention in the context of the publication of the Stern Review on the Economics of Climate Change (Stern et al. 2006). While this has helped generate more attention for an interdisciplinary debate with many important interventions by philosophers (such as Caney 2009, 2014; Gosseries and Meyer 2009; Davidson 2014), the focus here is mainly on the debates between economists.

‘The Stern Review on the Economics of Climate Change’ (Stern et al. 2006) was commissioned by the British government, and overseen by the economist Nicolas Stern. The Review recommends immediate, decisive and expensive measures, such as spending 1% of global GDP to counter the effects of climate change. It has been met with a mixed reception, questioning both recommendations and methodology of the review. The methodological controversies are focused on the role of intergenerational discounting. The Stern Review states that ‘it is hard to see any ethical justification for [discounting the welfare of future generations]’ (Stern et al. 2006, p. 35). Indeed, the issue of how to value costs and benefits to future generations has been key in the discussions that followed the publication of the review. Please see Quiggin (2008) for a compelling and engaging overview of the various reactions and debates. In the following, the focus s on analysing the role of values without engaging in a full review of all the aspects of the debate.

In his survey, Weitzman (2007, p. 705) cites numerous commentators who argue ‘that the strong conclusions of the Review are driven mainly by the low assumed discount rate…’. Likewise, Dasgupta (2006, p. 2) maintains: ‘The strong immediate action on climate change advocated by the authors is an implication of their views on intergenerational equity; it isn’t so much by the new climatic facts the authors have stressed.’ Hence, the way in which impacts on future generations are discounted in current decisions about environmental problems has been important in the critical reaction to the Review amongst economists. At the same time, it is important to consider that the discount rate was just one part of a broader methodological criticism of the Stern Review, for instance by Tol (2006), Tol and Yohe (2006, 2009). The focus in the following is on the debates about discounting in climate economics alone.^3^

The immediate reactions to the Stern Review with regards to the issue of time discounting by some prominent reactions can be summarized by the following claims: that the Stern Review made strong (and mistaken) ethical assumptions (such as argued especially by Dasgupta 2006, Weitzman 2007), and that it deviated from how other economists use social discount rates in intergenerational decision-making in various ways, (especially by Nordhaus 2007, Tol 2006, as well as Tol and Yohe 2006, 2009). These responses have created a lively debate, with several replies by Stern and his collaborators (most prominently, Dietz et al. 2007a, b, c, d, as well as Dietz and Stern 2008).

Accordingly, the ensuing debate about time discounting in climate change is marked by an explicit attention to ethical considerations. Most economists that reacted to the Stern Review directly argued that it was bringing in ethical considerations into economic policy analysis in the wrong way. The subsequent literature in economics has gone on to conduct discussions that are phrased explicitly in terms of ethics and value. The contributions by Atkinson et al. (2009), Asheim (2010), Buchholz and Schumacher (2010), Roemer (2011), Dasgupta (2011), Schneider et al. (2012), as well as Fleurbaey and Zuber (2014) make direct references to values or signal moral and ethical debates, by employing concepts such as ‘fair’, ‘equity’, ‘inequality’, ‘welfare’, ‘ethics’, ‘distribution’, and ‘utilitarianism’. Many of them go on to use ethical concepts in their arguments, or make them a key target. It is thus clear that these contributions are explicitly discussing normative questions: these authors are engaged in an ethical debate with each other.

### Values in the Climate Ethics Discounting Debate

Let us take stock. Firstly, it was observed that time discounting itself is an ethical problem, and secondly, it was observed that values indeed play an important role in the discussions about time discounting in climate economics more specifically. This section turns to a third observation: that the prevalent methodology of time discounting used in climate economics, that of the social discount rate, entangles facts and values. That is to say, referring to values is inescapable in that methodology.

Consider again the social discount rate and its three components r = delta + eta * g, where delta is the pure time preference, eta is the elasticity of marginal consumption (the percentage change in welfare derived from a percentage change in consumption or income), and g the growth rate of per-capita consumption or income over time. The interpretation of all these three components is highly complex, and goes beyond what can be reasonably covered here.^4^ What is interesting for the present purpose, however, are not so much the actual problems of interpretation that economists discuss but the role that values play.

Consider Stern et al. (2006). They assume 0.1% for delta, with pure time preference assumed to be 0, and a measure of the possibility of the extinction of humankind of 0.1%. They further assume the value of 1 for eta (meaning that 1 Dollar is worth 10 times more to someone with one-tenth of the income. Usual estimates of are between 0.5 and 1.2, varying per region and time). Finally, they also assume growth rates between 1.5 and 2% (this differs over the economic scenarios). Since some of the values for the parameters differ over economic scenarios, and regions and times, there is an implied discount rate of between 1.6 and 2.1% in the Stern Review (Quiggin 2008), which critics like Weitzman (2007) and Nordhaus (2007) found too low, when compared to both market rates and assumptions made in other analyses. Furthermore, there is strong disagreement over the assessments that implied in the different components between Stern et al. (2006) on the one hand and Dasgupta (2006), Weitzman (2007) Nordhaus (2007), Tol (2006), as well as Tol and Yohe (2006, 2009).

Stern et al. (2006) have initially viewed delta as a value judgement about the importance of future generations. They assumed it to be 0, as they did not think it made sense to assume that future generations are of less value than the present one, only to in addition include an assessment of the possibility of the extinction of humankind, which gave a delta of 0.1%. Almost all of the authors that have immediately reacted to Stern et al. (2006) are critical about assuming 0 for delta, as that does not reflect the time preference of individuals on markets. For the elasticity of marginal consumption, it is important to note that it simultaneously reflects concepts such as aversion to risk, spatial inequality and intertemporal inequality. It thus carries more than one meaning and relates to different parts of the model. Here, Tol and Yohe (2006, 2009) as well as Nordhaus (2007) are especially critical of the choices of Stern et al. (2006).

Many concepts discussed in the interpretation of the parameters that were referred to in the preceding paragraph are linked to value judgements. It should thus be clear from the above discussion that the interpretation of parameters in the social discount rate cannot be conducted purely in terms of facts, because it is impossible to properly distinguish between value judgements and factual judgements for all the parameters. While it may be possible to argue for such a strong distinction in the case of g (as one could maintain that projections of growth rates are a matter of value-free economics), this is much harder for eta and delta, the other two components. For eta, it is impossible to maintain because it has several functions in the model, some of which will pertain to ethical value judgements (such as the spatial and intertemporal equality). For delta, while one can attempt to discuss some aspects of it with regards to facts (such as the proposal to interpret it as a scientific judgement about the likelihood of extinction of humankind), even denying that one wants to make an ethical judgement such as Stern et al. (2006) are making turns out to be making one. Ultimately, this relates back to the fact that time discounting is, at bottom, an ethical problem. The methodology of the social discount rate, while designed to distinguish between different aspects of time discounting, does not provide a framework in which facts and values can be separated. (See Baum 2009 for a discussion that makes this point in more detail, but with different terminology.) Time discounting in economics is thus an obvious case for values being central to economic theorising and policy-making. It is an instance of economists freely making explicit ethical statements (concerning theory, measurement, practical application, policy-making, amongst others).

The picture of time discounting methodology entangling facts and values clashes with the idea of separation of facts and values that is upheld in science (Putnam 2002; Douglas 2009). Betz (2013) has recently reviewed and critically examined discussions about the ‘value-free ideal’, distinguishing between different philosophical critiques of it, and maintained that it can be defended. The value-free ideal is thus itself a value-laden position, but one that concerns the methodology of scientific inquiry (thus it can be said to be a meta-value). It is important to stress that the value-free ideal does not state that all of science (or, in our case economics) *is* value-free. Rather, the value-free ideal maintains that there are areas of the sciences that are value-free, and that scientists should strive to make more of science value-free. This has also been discussed concerning economics. Partha Dasgupta (an economist) on the one hand and Hilary Putnam and Vivian Walsh (two philosophers) on the other hand have conducted an important debate about facts and values in economics (Dasgupta 2005, 2007, 2009, Putnam and Walsh 2007, 2009, 2012). While Dasgupta has maintained that there can be some parts of economics that are value-free, Putnam and Walsh have pointed out that there are also value judgements involved in non-ethical statements, such as defining the meaning of a technical term: for example, a shared understanding of what the notion of ‘unemployment’ means contains value judgements about the kinds of situations that the term should refer to.

It is therefore of significance that in the case of time discounting in climate economics, economists have given up the value-free ideal. They not only explicitly acknowledge that they conduct ethical debates, which has been observed earlier. In this section it was shown that the methodology of time discounting does not allow economists to separate facts from values, as all parameters of the social discount rate can be argued to contain at least some value judgements.

## Methodological Values for Time Discounting

### From Values and Facts to Ethical and Scientific Judgements

Let us now turn to show that climate economists have adopted a methodology that not only entangles facts and values, but that there is an even deeper entanglement, which will be called mixing ethical and scientific judgements. The latter distinction entails a much weaker requirement than the requirement to disentangle facts and vales. First, the weaker distinction between ethical and scientific judgements will be explained, and contrasted to the stronger distinction between facts and values. Then, it will be observed that the weaker requirement of disentangling ethical and scientific judgements is also not met in the methodology of time discounting.

Analysing the fact-value debate in economics, Su and Colander (2013, p. 18) have pointed out that Putnam and Walsh ‘missed Dasgupta’s pragmatic arguments about how to move forward in tentatively separating positive truths from normative rules’. This characterisation of the debate is helpful: on one side of the debate, there are two philosophers who point out that value judgements can be found more often than one may think at first, and on the other side is an economist who acknowledges this, but still finds some sort of distinction between facts and values helpful. Facts and values can often be entangled, some of the times more deeply than at other times. In time discounting, it should not be very surprising that facts and values are deeply entangled, as it is an ethical problem itself. Given that facts and values are entangled, more specific questions can be asked, such as how deeply they are entangled, and how scientists should deal with this.

Consider the following proposal to distinguish between two kinds of judgements that are closely related to the fact-value distinction: *ethical* and *scientific* judgements. Firstly, one can maintain that there are explicitly ethical judgements, which are value-laden and normative. Many, if not all, commentators in the debates about discounting would, for instance, agree that there are such ethical judgements involved in the Ramsey parameters of delta and eta. Secondly, one can maintain that there are also scientific judgements, which aim at identifying facts about the economy (scientific judgements about the climate can also be included in this). Many commentators in the discounting debate would for instance characterise judgements about g in the Ramsey equation as a scientific judgement. Such scientific judgements will also inchoate many kinds of value judgements (about which models and measurements to use, which theories to accept, and what kind of data to collect, or even about how useful society may find a particular approach).

The distinction between ethical and scientific judgements is thus related to, but subtly different from, the distinction between facts and values. Recall that the distinction between statements of facts and values is related to *content*. That is, those who maintain facts and values can be sharply separated, support the idea that a statement either states a fact or a value, and those who deny the distinction say that there are no pure factual statements. The distinction between ethical and scientific judgement, however, is one of the primary *aims* of the judgement that a researcher or scientist is making. An ethical judgement aims to express what is right, or good, or virtuous. Now, an ethical judgement may also make use of facts assembled by scientists, but the aim of it is to pass judgements of value. A scientific judgement aims to say what was, is, or will be fact. As such, it will also make implicit or explicit use of scientific, ethical, cultural, and social values along the way, but the primary aim of the scientific judgement is to identify facts. The distinction between ethical and scientific judgement thus does not presuppose that we can successfully distinguish between facts and values. It also does not merely reformulate the distinction between facts and values. What it captures is that the *goal* of asking a question or making a judgement can be different. For instance, ethicists have the primary aim of making ethical judgements and thus clarifying values, and scientists have the primary aim of making scientific judgements and thus clarifying facts. Crucially, recognising that such difference in goal exists does not rest on endorsing any position concerning whether it is possible to sharply distinguish between facts and values.

The distinction between ethical and scientific judgements is broadly consistent with Su and Colander’s (2013) take on the fact-value debate. Indeed, as it is a much weaker distinction, it has room to acknowledge both the Putnam and Walsh point about the pervasive role of values and the pragmatic perspective of Dasgupta. Indeed, one can recover either position by starting with the distinction between ethical and scientific judgements and adding specific assumptions. If one wants to recover Putnam and Walsh’s position concerning facts and values, one needs to assume that all scientific judgements necessarily contain assertions about values. If one wants to recover Dasgupta’s position, one needs to make two assumptions: first, one needs to assume that economists strive to make scientific judgements. Second, one needs to maintain that while critical ingredients of scientific judgements (such as definitions, concepts, distinctions, and delineations) may be argued to also relate to or build on values in some way, it is more helpful to reserve the term ‘values’ for explicit ethical judgements. Note that doing so does not mean to endorse either position. Rather the aim is to illustrate that the distinction between aiming for ethical or scientific judgements allows for recovering a variety of positions in the fact-value debate by making additional assumptions in the way just demonstrated.

Applying the distinction between ethical and scientific judgements reveals further issues with discounting methodology. While delta is seen as an exclusively ethical judgement about either time preference or the moral importance of future generations by many commentators, it does (at least for Stern and collaborators) also include a scientific judgement (that captures the probability of survival of humankind). Likewise, even if eta is seen as an ethical judgement, it does depend on a lot of factors in the economic models that are scientific judgements made by economists. Only g seems to be a relatively straightforward scientific judgement. (To be sure, there are many ethical arguments to be had about the growth rate, and about the desirability of growth. But the decision which growth rate prediction to use in the Ramsey equation can be characterised as a scientific judgement.) Thus, the Ramsey methodology of discounting is not employed in a way that allows us to distinguish between those judgements that are primarily ethical and those that are primarily scientific.

Why is adopting a methodology that does not allow do distinguish between ethical and economic (scientific) aims problematic? One of the most important reasons is that it makes it harder to deploy with precision the respective tools that ethicists and economists have developed for answering questions of the respective kind. However, the present article does not intend to give full justification to the claim that failing to distinguish between ethical and scientific judgements is problematic; rather, the main aim is to record the observation that the time discounting methodology adopted in climate economics has not been used in a way that respects this distinction.

### Values and the Methodology of Time Discounting

What sort of methodological lessons should be drawn from the four observations made in this article? It seems that there are two routes that can conceivably be taken. The first one would be to completely abandon the social discount rate methodology in favour of a framework that might be less prone to fact-value entanglements, and only to investigate time discounting in as far it can be captured in an mathematical framework, like that of axiomatised time preference, that allows for a numerical representation of time preference as discounted utility.^5^ Call this the ‘representation paradigm’.

The representation paradigm essentially says that concepts in economics should be ultimately traceable to a framework of numerical representation, in which conditions can be given by axioms, and any quantities or numbers are numerical representations (following, for instance, the mathematical frameworks of the representational theory of measurement, reviewed in Heilmann 2015). The representation paradigm has been popular in economic theory, via rational choice theory (both in terms of individual decision theory, but also in game theory). The difficulty with adopting the representation requirement is that it leaves economics without a method for social discounting, as the time preference framework, and other axiomatic frameworks that have been put forward so far, lend themselves to individual discounting, and much less so to social discounting. That is because axiomatic frameworks for time discounting have been formulated in terms of discounted utility, which is conceptualised primarily as capturing individual decision-making. Moreover, even though the time preference framework has been generalized a fair bit in mathematical terms (e.g. Bleichrodt et al. 2008), it is conceptually not rich enough to discuss a variety of motivations for time discounting. While the representation paradigm may be attractive from a foundational point of view, it does not seem to be flexible enough to handle the methodological demands from climate economics. Thus, there does not seem to be a ready solution for time discounting in climate economics from within the representation paradigm.

What can be said, however, is that a second route should be adopted, possibly within the social discount rate methodology, or as a constructive critique of it: that of distinguishing between ethical and scientific judgements concerning time discounting. Note that this is a much weaker requirement than separating facts and values, or requiring that non-epistemic values be kept out of a certain area of scientific enquiry. It is consistent with making scientific judgements to acknowledge that non-epistemic values play a role, and that there hence need not be (or cannot be) a strict separation between facts and values.

To illustrate this idea, consider a brief example that is not related to the topic at hand. Consider the case of trying to achieve more diversity with regard to certain social categories in hiring (think of age, gender, cultural, or ethnic background). There will be a number of judgements involved in making this operational, such as determining on which social categories more diversity is to be achieved. Presumably, such a determination will involve scientific judgements, such as a review of relevant evidence from demography, social science, and psychology. While ultimately the judgement about which social categories to invoke, and in what way, will be value-laden, and should probably be called an ethical one, there will be various scientific judgements that serve as an input. Without assuming that facts and values can be separated in any of the steps that lead up to such a decision, it still is beneficial to delineate certain scientific judgements, such as what evidence from the sciences should or should not play a role (while being aware that non-epistemic values may enter into such scientific judgements).

In climate economics, the idea of separating ethical and scientific judgements can be used in this way. A straightforward requirement would be to demand that any component of the social discount rate ought to either have a primarily scientific or ethical interpretation. That would probably mean to severely re-design the application of the social discount rate methodology. Analogous to the above example of diversity in hiring, a weaker requirement would be to spell out in more detail which scientific and ethical judgements should be made, and how they relate to, and depend on, each other.

A more poignant application of the idea that ethical and scientific judgements should be distinguished is this: it can be used to ask for more clarification between the judgements that are inherent in the general economic analysis (such as the integrated assessment models, or the cost–benefit analysis inherent in them) on the one hand, and time discounting on the other hand. As it stands, debates about time discounting in climate economics are hard to adjudicate because it is not clear to what extent there are judgements about, for instance, uncertainties related to the existence of humankind, risk aversion, and inequality already inherent in the broader modelling frameworks. In a word, there are many interactions between the ethical and scientific judgements within the time discounting methodology and those within the cost–benefit or welfare analysis. Consider the overview by Robert and Zeckhauser (2011), who discuss climate economics as a case of normative policy analysis. Tellingly, in their overview of ‘Climate policy: Sources of disagreement’ (Robert and Zeckhauser 2011, Table 2 on p. 621), there are both ‘positive’ and ‘normative’ disagreements recorded for time discounting.

As a more specific example, take the interpretation of delta in Stern et al. (2006), and the fact that it includes a probabilistic assessment of the possibility of the extinction of humankind. Now, any sensible framework of intergenerational decision-making will also include an scientific assessment of future scenarios, and record the uncertainties and probabilistic judgements accordingly. The challenge for using the social discount rate methodology, and interpret delta in this way is then to rule out that there is any interaction, dependence or ‘double-counting’ of the uncertainties elsewhere in the model. Hence, one needs to assume that it is possible to provide a separate scientific assessment of the possibility of the extinction of humankind, and have it be neutral with regards to other scientific assessments. Likewise, the values for eta and g depend heavily on the whole range of ethical and scientific assessments that go into the assumptions of welfare and growth rate measurement. Whether this is realistic remains to be seen. All that one may be able to do within the social discount rate methodology is to be more explicit with regards to these relations.

Such a stance seems to echo more general perspectives that have been offered with regards to climate economics methodology. For instance, Heal (2009) offers a wide-ranging review of work in climate economics and offers perspectives for the future of the field. His arguments can be summarized as proposing to discuss time discounting still within the social discount rate methodology, but make both ethical and scientific judgements more explicit, as well as their relations to the wider frameworks of welfare and cost–benefit assessment. Nelson (2008) offers a more fundamental critique on such recommendations from the perspective of values in science, calling for a more thorough evaluation of the underlying modelling assumptions in the models of climate economics.

Concerning time discounting in climate economics, the perspective of separating between ethical and scientific judgements thus offers a highly critical stance towards the social discount rate methodology and its conduciveness to advancing the debates. As Quiggin (2008, p. 203) puts it in his comment: ‘In analysing such problems we are pushing economic analysis to its limits.’ One contribution of this article is to achieve a better understanding of why these limits seem to be quite severe, and offer the entanglement of facts and values as well as ethical and scientific judgements as one plausible explanation, or at least a contributing factor to it.

## Conclusions

This article has argued that firstly, time discounting is an ethical problem in and of itself. Secondly, it was shown that time discounting in climate economics in general is a case of values being central to economics and economists freely making explicit ethical statements. Thirdly, it was argued that time discounting methodology in climate economics is a case of deep theoretical entanglement between facts and values. Fourthly, it was argued that the methodology of time discounting also entangles ethical and scientific assessments.

From a methodological perspective, and especially one that focuses on the role of values in science, the prospects for time discounting in climate economics do not look promising. What this perspective offers, however, may also be an explanation for why the debates concerning time discounting in climate economics are so entrenched. The constraints and limitations of the framework in which economists discuss these issues seem to be, on the picture that has been advanced in this article, one important reason for why the time discounting issue is pervasive.

## Footnotes

1 For r = 0.02 and t = 10, the formula D(t) = [1/(1 + r)]^t yields D(10) = 0.82, which in turn yields roughly 37 Million Euros when multiplied by 45 Million Euros.

2 This is not to deny that there is a growing literature that considers alternative methodologies, such as in Gollier (2012), Medvecky (2012), Traeger (2014), Fleurbaey and Zuber (2014), Davidson (2014) and Aalbers (2013) who explore discounting beyond the received frameworks that are the main focus of this article.

3 Hansen (2011) analyses the interdisciplinary aspects of these exchanges.

4 For a revealing overview of the conceptual and interpretative disagreements between economists about the parameters of the social discount rate, see the survey by Drupp et al. (2015).

5 See also the discussion in the first section. Axiomatising time preference, in turn, allows conducting empirical work, such as eliciting time preferences of individuals.
